# Sportrictiosis

**Published:** 1913-01

**Authors:** 


Sportrichosis.—Hamburger. (*Journal Amer. Med. Assn.*, Nov. 2, 1912.) reports a case and states that it is highly probable that this is a wide-spread, prevalent disease, particularly in country and farming districts, and that many cases now diagnosed as tuberculosis, syphilis, glanders, blastomycosis, actinomycosis, etc., are really unrecognized sporotrichosis.

The disease occurs more often in men between 15 and 45, usually in country districts. Ordinarily, there is a history of trauma. The incubation is slow and there follows a slowly progressive, ascending infection, following the course of the deep lymphatics, with the production of characteristic small, hard, round, subcutaneous nodules, which break down to form cold abscesses or cutaneous ulcers.

The disease lasts from three weeks to eighteen months, with little or no pain or temperature and little or no effect on the general health.

The Sporotrichium appears as a growth on 2% glucose agar, at room or incubator temperature, in eight days. It is a branching, septate mycelium with pear-shaped spores.

